# A pro-inflammatory environment in bone marrow of Treg transplanted patients matches with graft-versus-leukemia effect

**DOI:** 10.1038/s41375-023-01932-x

**Published:** 2023-06-07

**Authors:** Francesco Guardalupi, Carlo Sorrentino, Giulia Corradi, Raffaella Giancola, Stefano Baldoni, Francesca Ulbar, Bianca Fabi, Rosa Andres Ejarque, Jessica Timms, Francesco Restuccia, Stella Santarone, Patrizia Accorsi, Paolo Sportoletti, Filomena De Falco, Emanuela Rosati, Alessandra Carotti, Franca Falzetti, Andrea Velardi, Massimo Fabrizio Martelli, Shahram Kordasti, Antonio Pierini, Loredana Ruggeri, Mauro Di Ianni

**Affiliations:** 1 Department of Medicine and Aging Sciences, University of Chieti-Pescara, Chieti, Italy; 2 Department of Oncology Hematology, Pescara Hospital, Pescara, Italy; 3 System Cancer Immunology, Comprehensive Cancer Centre, King’s College London, London, United Kingdom; 4 Department of Medicine and Surgery, University of Perugia, Perugia, Italy

**Keywords:** Translational research, Bone marrow transplantation, Immunotherapy

## To the Editor:

In HLA haploidentical transplantation, T regulatory cells (Tregs) co-infused with conventional T cells (Tcons), protect against graft-versus-host disease (GvHD) while maintaining the graft versus leukemia effect (GvL) [1–3]. GvHD prevention might be due to the CTLA-4 dependent downregulation of CD80/CD86 on dendritic cells (DCs) [4] while the GvL effect could be linked to the ability of Tregs to reduce the expansion of T cells, but not their activation [5]. Here, we described the selective localization of the CD161^+^ Tregs in the bone marrow of transplanted patients. This population, already described as capable of producing pro-inflammatory cytokines [6], may be fundamental for developing a microenvironment capable of maintaining a GvL effect. 20 patients were recruited (Supplementary Methods) and received an haplo-HSCT with 2 × 10^6^/kg Tregs, 1 × 10^6^/kg Tcons and a megadose of CD34^+^ cells [1–3]. Peripheral blood (PB) and bone marrow (BM) samples were collected at 1, 3, 6, and 12 months after transplantation.

BM- and PB-DCs were analyzed on BD FACS Lyric System (BD Biosciences, La Jolla, CA), using the antibodies listed in Supplementary Table 1. DCs were sorted by using anti-CD123 (for plasmacytoid DCs, pDCs) and anti-CD11c Abs (for myeloid DCs, mDCs) (Supplementary Table 1) and analyzed, by real-time RT-PCR, for the expression of *Indoleamine 2,3-Dioxygenase 1* (*IDO-1*), *Interleukin (IL)-6*, *IL-10*, *Programmed Death Ligand 1* (*PD-L1*) and *Transforming Growth Factor Beta 1* (*TGFB1*) (Supplementary Table 2). BM- and PB-CD11c^+^ DCs were collected from patients and co-cultured with autologous CD3^+^ cells. To generate CD161^+^ Tregs, cells were purified from PB of healthy donors (HDs), cultured with IL-2, IL-6, and TGF-β and used for functional assays (see Supplementary Methods).

Student *t*-test and ANOVA analysis were performed, followed by the Tukey and Sidak’s multiple comparison test. All statistical tests were evaluated at an α level of 0.05, using Stata, version 13 (StataCorp, College Station, TX, USA; RRID:SCR_012763) and GraphPad Prism software, version 9 (RRID: SCR_002798, San Diego, CA, USA).

**Fig. 1:**
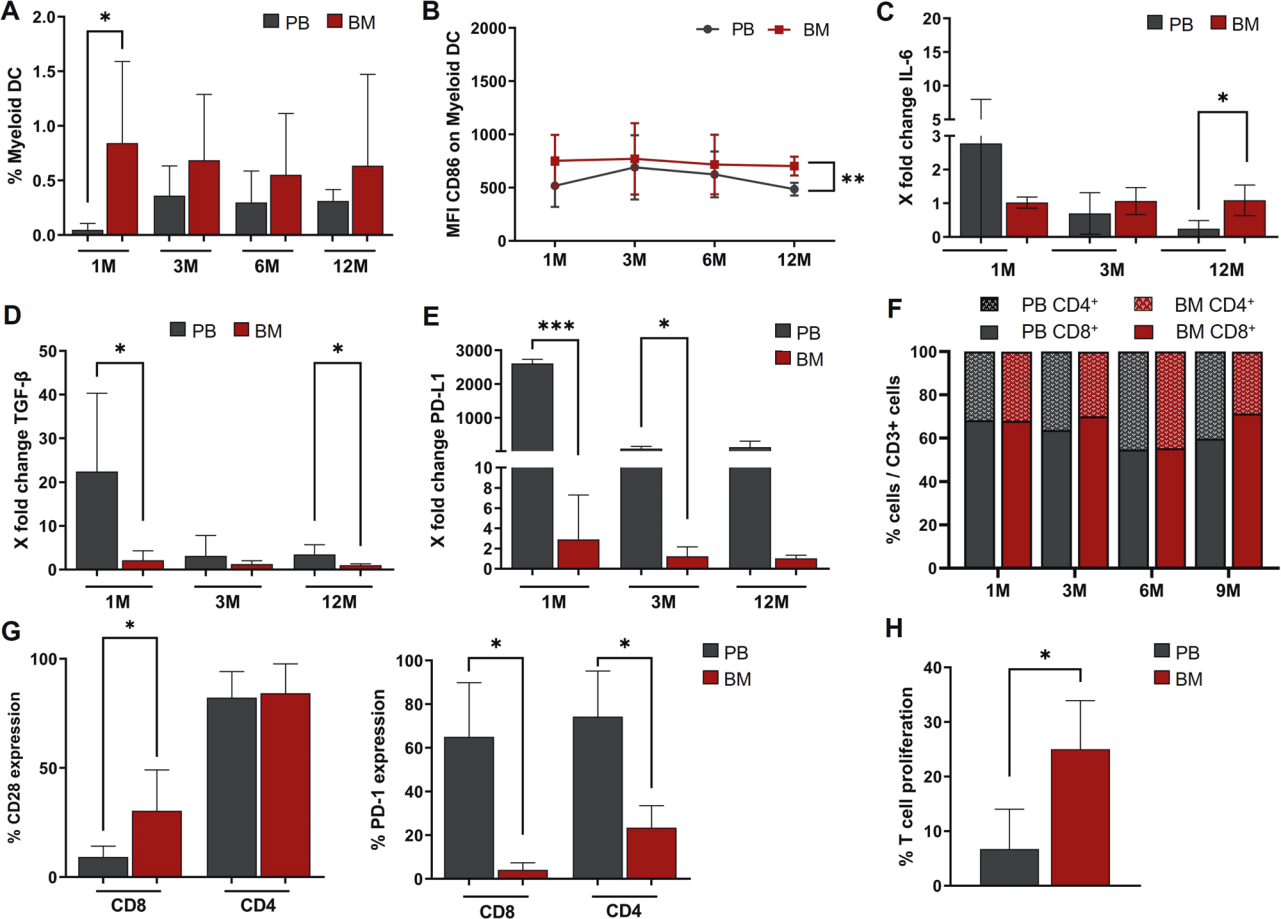
Flow cytometry and real-time RT-PCR analysis of DCs, derived from BM or PB of transplanted patients. Flow cytometry analysis of CD4^+^ and CD8^+^ T cells and mixed culture of CD3^+^ T cells with BM- or PB-mDCs. **A** The mDC percentage was higher in BM than in PB, in the first month after transplant (^*^Student’s *t*-test: *p* < 0.05). **B** BM-derived mDCs expressed higher levels of the co-stimulatory receptor CD86 (showed as MFI values), compared to PB-derived mDCs (^**^Student’s *t*-test: *p* < 0.01). **C**, **D** Real-time RT-PCR showed the gradual decrease of *IL-6* and *TGF-β* expression, after transplantation, in PB-mDCs, while their levels in BM-mDCs remain stable. (^*^Student’s *t*-test: *p* < 0.05). **E** Real-time RT-PCR showed significantly higher *PD-L1* levels in PB-mDCs compared to BM-mDCs (^*^Student’s *t*-test: *p* < 0.05; ^***^Student’s *t*-test: *p* < 0.001). All data (**A**–**E**) are represented as mean ± SD. **F** The percentages of CD3^+^, CD4^+^ and CD8^+^ T cells were comparable (Student’s *t*-test: *p* > 0.05) between BM and PB and among early and late follow-up phases (ANOVA: *p* > 0.05). Data are represented as mean. **G** Left Panel. BM-derived CD8^+^ T cells displayed a higher expression of the co-stimulatory receptor CD28 than PB-derived CD8^+^ T cells (30.3% ± 18.8 vs 9.2% ± 4.9; ^*^Student’s *t*-test: *p* < 0.05). Data are represented as mean ± SD. Right Panel. The expression of the immune checkpoint inhibitor PD-1 was significantly higher in PB-derived CD4^+^ (69% ± 29 vs 24% ± 11; ^*^Student’s *t*-test: *p* < 0.05) and CD8^+^ (65% ± 25 vs 4% ± 3; ^*^Student’s *t*-test: *p* < 0.05) T cells than in BM-derived T lymphocytes. Data are represented as mean ± SD. **H** CD3^+^/CFSE^+^-mDCs co-cultures showed a T cell proliferation rate that was significantly higher when T cells were cultured in presence of BM-mDCs (25% ± 7.2 vs 6.7% ± 8.7; ^*^Student’s *t*-test: *p* < 0.05). Data are represented as mean ± SD of 3 independent experiments.

The enrolled patients’ clinical outcome (GvHD; Relapse rate; TRM) was in line with the published data [3]. All enrolled patients have full donor chimerism. Flow cytometric analysis, revealed that percentage of mDCs was significantly higher in BM (range: 0.16–2.03%) than in PB (range 0–0.18%) samples in the first month after transplantation (Fig. 1A). BM-derived mDCs expressed higher levels of the co-stimulatory receptor CD86 (MFI ranges PB: 411–548; BM: 603–859) 12 months after transplantation (Fig. 1B). No differences emerged in pDCs. RT-PCR showed that, in PB-mDCs, IL-6 and TGF-β expression gradually decreased after transplantation, while it remained almost unchanged in BM-mDCs (Fig. 1C, D). Because of this, after twelve months from transplantation, IL-6 secretion in PB-mDCs was significantly lower than in BM-mDCs (Fig. 1C). On the other hand, the expression of the immune-checkpoint regulator PD-L1 remained extremely high in PB-mDCs, compared with BM-mDCs (Fig. 1E). This indicates the presence of an immunosuppressive signature in PB-mDCs. The expression of the costimulatory molecules and immune checkpoints were similar to Carenza et al. [7].

The analysis of the T cell compartment showed that, although the percentages of CD3^+^, CD4^+^ and CD8^+^ cells in BM and PB were comparable (Fig. 1F), BM-derived CD8^+^ T cells, at 3^rd^ month, displayed a higher expression of the co-stimulatory receptor CD28, compared to PB-derived CD8^+^ T cells (30.3 ± 18.8 vs 9.2 ± 4.9; Student’s *t*-test: *p* < 0.05) (Fig. 1G Left Panel). Furthermore, the expression of the immune checkpoint inhibitor PD-1 was significantly higher, in the 3^rd^ month, in both PB-derived CD4^+^ (69% ± 29 vs 24% ± 11) and CD8^+^ (65% ± 25 vs 4% ± 3; Student’s *t*-test: *p* < 0.05) T cells than in BM-derived T lymphocytes (Fig. 1G Right Panel). T cells from both BM and PB, express very low levels of the T cell exhaustion marker TIM3 (CD8^+^: 1.93% ± 1.46 in PB vs 1.87% ± 1.45 in BM; CD4^+^: 1.10% ± 1.22 in PB *vs* 5.43% ± 5.35 in BM), with no significant differences between the two compartments (Student’s *t*-test: *p* > 0.05). The rescue of CD8^+^ cells by PD-1-targeting is CD28-dependent and the T cell costimulatory receptor is a primary target for PD-1 inhibition [8].

To investigate the role of mDCs in the activation/inhibition of T cells in the BM or PB microenvironment of transplanted patients, we performed mixed lymphocyte cultures in which CD3^+^ T cells (obtained from patients at the 3^rd^ month post-transplant) were cultured together with autologous BM- or PB-derived mDCs. These in vitro studies demonstrated that T cell proliferation was significantly higher (25% ± 7.2 vs 6.7% ± 8.7; Student’s *t*-test: *p* < 0.05) when T cells were cultured in the presence of BM-derived mDCs (Fig. 1H).

**Fig. 2:**
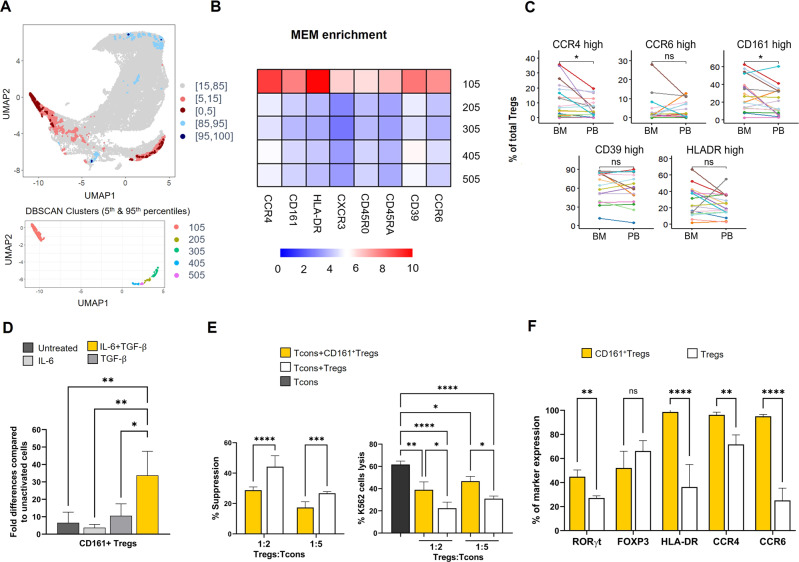
Flow cytometric analysis of CD161^+^ Treg cells derived from BM or PB of transplanted patients, in vitro generation of CD161^+^ Treg cells with related functional experiments. **A** Upper panel. UMAP plot with T-REX analysis of bone marrow and peripheral blood Tregs based on a statistical threshold of ≥ 95% change in cell phenotypes. Pink and red colour denote regions of phenotypic change identified by T-REX with a higher representation in bone marrow samples. Blue areas are enriched in peripheral blood. (Light colours: 85–95% change, dark colours: ≥95% change). Lower panel. DBSCAN clustering for the areas of ≥95% change. **B** Heatmap showing MEM enrichment of Treg markers in DBSCAN clusters. **C** Percentage of Tregs with high expression of CCR4, CCR6, CD161, CD39, HLA-DR in bone marrow and peripheral blood samples. Each patient is represented by a line. *p < 0.05 (two tailed paired Wilcoxon test). **D** PB-derived Tregs (CD4^+^CD25^high^CD127^-/low^), treated with both IL-6 and TGF-β, showed, after 10 days of culture, a significant higher expression of CD161 (One-way ANOVA, Tukey HSD test: *p < 0.05, **p < 0.01), if compared with cells untreated or treated with IL-6 or TGF-β alone. Untreated = cells only activated with T Cell TransAct™ and IL-2. Data are represented as mean ± SD of 3 independent experiments. **E** The graphs show the functional activity of CD161^+^ Tregs. Left panel. Suppression activity of CD161^+^ Tregs and expanded Tregs, referred as Tregs, on the proliferation of Trans Act stimulated-Tcons in a mixed lymphocyte reaction performed for 4 days. Data are presented as mean ± SD of 3 independent experiments (Two-way ANOVA, Sidak’s multiple comparison test; ****p < 0.0001; ***p = 0.0009). Right panel. Killing of the K562 cell line by Tcons, ratio Effector:Target 1:20. Where indicated, Tcons were co-coltured with CD161^+^ Tregs and Expanded Tregs, referred as Tregs, at different Tregs:Tcons ratio, 1:2 and 1:5 respectively (mean ± SD of 3 experiments; One-way ANOVA, Tukey’s multiple comparison test: Tcons alone vs + CD161^+^ Tregs ratio 1:2 **p = 0.001; vs + Tregs ratio 1:2 ****p < 0.0001; vs + CD161^+^ Tregs ratio 1:5 *p = 0.021; vs + Tregs ratio 1:5 ****p < 0.0001; + CD161^+^ vs Tregs ratio 1:2 *p = 0.011; + CD161^+^ vs Tregs ratio 1:5 *p = 0.015). **F** Flow cytometric analysis of the expression of RORγt, FOXP3, HLA-DR, CCR4, and CCR6 on CD161^+^ Tregs, defined as CD45^+^CD3^+^CD4^+^CD25^high^CD127^-/low^CD161^+^ and Expanded Tregs, referred as Tregs and defined as CD45^+^CD3^+^CD4^+^CD25^high^CD127^-/low^ (mean ± SD of 3 experiments; Two-way ANOVA, Sidak’s multiple comparison test, **p = 0.005; ****p < 0.0001).

These data show that haploidentical transplantation, associated with Tregs/Tcons immunotherapy, promotes the reconstitution of DCs with an activating phenotype in the BM and a tolerogenic activity in the PB. Moreover, we analyzed flow cytometric data from BM and PB samples, using the T-Rex (Tracking responders expanding) algorithm. This unsupervised machine learning analysis led us to identify, a specific cluster of Treg cells, characterized by CCR4, CD161 and HLA-DR positivity, in the BM of transplanted patients (Fig. 2A–C), starting from the first months after the transplant. This population [6], has similar phenotypical characteristics to other subpopulations of Treg cells, but can be induced to express the phenotypic profile of Th17 cells in the presence of inflammatory cytokines. In particular, these Treg cells were found enriched within the inflamed joints of patients with inflammatory arthritis, compared to peripheral blood. However, in vitro analyses suggest that IL-17 production by this Treg-cell subpopulation with “IL-17 potential” is not necessarily accompanied by the acquisition of a proinflammatory function [9]. Interestingly, the percentage of CD161^+^ Tregs was not different between Acute-Myeloid-Leukemia- and Acute-Lymphoblastic-Leukemia patients (Supplementary Fig. 1C). Since the real-time RT-PCR showed a constant expression of *IL-6* and *TGF-β* in BM-mDCs, and it has already been demonstrated that both these cytokines can induce Th17 phenotype in naive T cells [10], we hypothesized that they could also play a role in the development of this Treg subpopulation. To confirm these hypotheses, we stimulated, in vitro, activated Tregs, purified from PB of HDs, with IL-6, TGF-β or both and found that the cells stimulated with the combination of the two cytokines showed, after ten days of culture, a significantly higher expression of CD161 (One-way ANOVA, Tukey HSD test: *p* < 0.01), if compared with cells untreated or treated with IL-6 or TGF-β alone (Fig. 2D).

Furthermore, to better investigate in vitro-generated CD161^+^ Tregs, we performed suppression and cytotoxicity assays using as control a population of expanded Treg cells derived from the same donors (*n* = 3 cases) [11, 12]. For the suppression assay, Tcons were cultured with autologous CD161^+^ Tregs or expanded Tregs at 1:2 and 1:5 Treg/Tcons ratios. After four days of culture, we showed a significantly lower suppressive activity mediated by CD161^+^ Tregs versus expanded-Tregs at both 1:2 and 1:5 Treg/Tcons ratios, respectively (28.7% ± 2.1; 17.3% ± 3.8; 44.2% ± 7.2; 26.7% ± 1.1; One-way ANOVA: ****p < 0.0001; ***p = 0.0009, Fig. 2E Left Panel). Conversely, the cytotoxicity assay performed 24-hours after co-culture of Tcons with both autologous CD161^+^ Treg or expanded-Treg cells (1:2 and 1:5 Tregs/Tcons ratios) in the presence of K562 tumor cell line (Supplementary Methods) demonstrated that CD161^+^ Tregs allow a significantly higher Tcons-mediated tumor killing compared to expanded-Treg cells at both 1:2 and 1:5 Treg/Tcons ratios, respectively (38.9% ± 7.0 vs 22.2% ± 5.5; 46.7% ± 4.3 vs 30.9% ± 2.3; One-way ANOVA: *p = 0.0108; *p = 0.015, Fig. 2E Right Panel). Overall, we found that CD161^+^ Treg cells suppress Tcons proliferation to a lower extent compared with expanded-Treg cells and favor a “permissive” Tcon-mediated killing capacity. These data suggest the role of CD161^+^ Tregs in mediating the skewing towards the GvL direction.

In addition, an in-depth analysis of CD161^+^ Treg phenotype showed a higher expression of CCR4, CCR6 and HLA-DR, compared to expanded Tregs. CD161^+^ Tregs also displayed a higher expression of RORγt, which is the transcription factor for Th17 lineage [13]. On the contrary, FOXP3 expression did not differ between the two cell populations, corroborating the evidence that they are both Tregs (Fig. 2F).

Further studies are needed to clarify whether CD161^+^ Tregs have only a permissive role towards GvL or whether they have an active role, carrying out a proinflammatory action in the bone marrow microenvironment. However, our results suggest that CD161^+^ Tregs originate from the interaction of Tregs with BM-derived mDCs [14], which produce IL-6 and TGF-β.

These findings are in agreement with what was observed by Ruggeri et al. [15] who hypothesize that the ability of donor Tregs to hamper GvHD, while maintaining GvL reactivity, is due to the prevalent CD45RO^+^CXCR4^-^ phenotype, which would prevent them from localizing and performing their suppressive activity in the bone marrow. Thus, the mechanism proposed by Ruggeri et al. could be prevalent in the first weeks post-transplant, while, in the following months, the maintenance of an effective GvL reactivity could be due to the development, in the bone marrow, of the CD161^+^ Treg population.

A more in-depth understanding of the interactions between Tregs and DCs could be the basis for future therapeutic strategies that will be able to selectively promote GvL in the BM and induce an immunosuppressive microenvironment in peripheral tissues, thus counteracting the onset of GvHD.

## Supplementary information

Supplemental Table 1

Supplemental Table 2

Supplemental Figure 1

Supplemental Legend

Supplemental Methods
