# Supplementary material for: A pro-inflammatory environment in bone marrow of Treg transplanted patients matches with graft-versus-leukemia effect
Source: Leukemia. 2023 Jun 7;37(7):1572–5. doi: 10.1038/s41375-023-01932-x (PMC10317833; doi:10.1038/s41375-023-01932-x)
Supplement: Supplementary file 1 — Supplemental Table 1 [file 41375_2023_1932_MOESM1_ESM.docx]

**Supplemental Table 1.** Reagents used in flow cytometry.

| **Antibody** | **Format** | **Research Resource Identifier (RRID)** | **Source** |
| --- | --- | --- | --- |
|  |  |  |  |
| CD3 | BV421 | AB_11152082 | BD Biosciences, Franklin Lakes, NJ, USA |
| CD3 | BV786 | AB_2738487 | “ |
| CD3 | PE-Cy7 | AB_2868767 | “ |
| CD4 | BV605 | AB_2744420 | “ |
| CD4 | APC-H7 | AB_1645478 | “ |
| CD4 | PE-Cy7 | AB_1727475 | “ |
| CD4 | FITC | AB_2868797 | “ |
| CD8 | BV786 | AB_2687487 | “ |
| CD11c | BV650 | AB_2869490 | “ |
| CD14 | BV786 | AB_2744287 | “ |
| CD16 | PerCP-Cy5.5 | AB_2868680 | “ |
| CD19 | PE-Cy7 | AB_2868769 | “ |
| CD20 | APC-H7 | AB_10561681 | “ |
| CD24 | PE | AB_395822 | “ |
| CD25 | BB515 | AB_2744340 | “ |
| CD25 | APC-H7 | AB_1645472 | “ |
| CD27 | APC-R700 | AB_2739074 | “ |
| CD27 | BB515 | AB_2744354 | “ |
| CD28 | APC | AB_398666 | “ |
| CD31 | PE-Cy7 | AB_2738348 | “ |
| CD38 | APC | AB_398599 | “ |
| CD39 | BV711 | AB_2738369 | “ |
| CD45 | V500-C | AB_2870390 | “ |
| CD45R0 | APC-H7 | AB_10562194 | “ |
| CD45RA | BV650 | AB_2738514 | “ |
| CD56 | BV711 | AB_2738043 | “ |
| CD57 | BV605 | AB_2632390 | “ |
| CD80 | PE | AB_396606 | “ |
| CD86 | BV421 | AB_11153866 | “ |
| CD95 | PE | AB_396027 | “ |
| CD123 | BV605 | AB_2732049 | “ |
| CD127 | APC-R700 | AB_2739099 | “ |
| CD127 | BB515 | AB_2738802 | “ |
| CD127 | BV421 | AB_11151911 |  |
| CD161 | PE | AB_396348 | “ |
| CD161 | BV605 | AB_2869531 | “ |
| CD183 | PE-Cy7 | AB_2033944 | “ |
| CD194 | PerCP-Cy5.5 | AB_1727416 | “ |
| CD196 | BV786 | AB_2738381 | “ |
| CD197 | PE-CF594 | AB_11153301 | “ |
| FOXP3 | Alexa Fluor**®** 647 | AB_1645411 | “ |
| HLA-DR | PE-CF594 | AB_11154415 | “ |
| HLA-DR | PerCP-Cy5.5 | AB_2868719 | “ |
| HLA-DR | V450 | AB_2716783 | “ |
| IgD | PE-CF594 | AB_11153129 | “ |
| RORγt | PE-CF594 | AB_2651150 | “ |
| PD-1  TIM3 | PE-Vio ® 770  VioBright®FITC | AB_2752076  AB_2654183 | Miltenyi Biotec, Bergisch Gladbach, D |
|  |  |  |  |
|  |  |  |  |
| **Reagents** | **Format** | **Research Resource Identifier (RRID)** | **Source** |
| Live/Dead™ Fixable Far Red Dead Cell kit | APC | - | Thermo Fisher Scientific, Waltham, MA, USA |
| 7AAD | PerCP | AB_2869266 | BD Biosciences, Franklin Lakes, NJ, USA |
