# Supplementary material for: A pro-inflammatory environment in bone marrow of Treg transplanted patients matches with graft-versus-leukemia effect
Source: Leukemia. 2023 Jun 7;37(7):1572–5. doi: 10.1038/s41375-023-01932-x (PMC10317833; doi:10.1038/s41375-023-01932-x)
Supplement: Supplementary file 2 — Supplemental Table 2 [file 41375_2023_1932_MOESM2_ESM.docx]

**Supplemental Table 2.** Primers* used for real-time RT-PCR.

| **Primer** | **Sequence (5’-3’)** |
| --- | --- |
|  |  |
| *GAPDH forward* | ATGGGGAAGGTGAAGGTCG |
| *GAPDH reverse* | GGGGTCATTGATGGCAACAATA |
|  |  |
| *IDO-1 forward* | GAATGGCACACGCTATGGAA |
| *IDO-1 reverse* | CAGACTCTATGAGATCAGGCAGATG |
|  |  |
| *IL-6 forward* | AACCTGAACCTTCCAAAGATGG |
| *IL-6 reverse* | TCTGGCTTGTTCCTCACTACT |
|  |  |
| *IL-10 forward* | TTACCTGGAGGAGGTGATGC |
| *IL-10 reverse* | GGCCTTGCTCTTGTTTTCAC |
|  |  |
| *PD-L1 forward* | TGCCGACTACAAGCGAATTACTG |
| *PD-L1 reverse* | CTGCTTGTCCAGATGACTTGGG |
|  |  |
| *TGFB1 forward* | GCAGCACGTGGAGCTGTA |
| *TGFB1 reverse* | CAGCCGGTTGCTGAGGTA |
|  |  |

*All primers were synthesized by Sigma-Aldrich Corporation (St. Louis, MO, USA).
