# Supplementary figures and images for: A pro-inflammatory environment in bone marrow of Treg transplanted patients matches with graft-versus-leukemia effect
Source: Leukemia. 2023 Jun 7;37(7):1572–5. doi: 10.1038/s41375-023-01932-x (PMC10317833; doi:10.1038/s41375-023-01932-x)

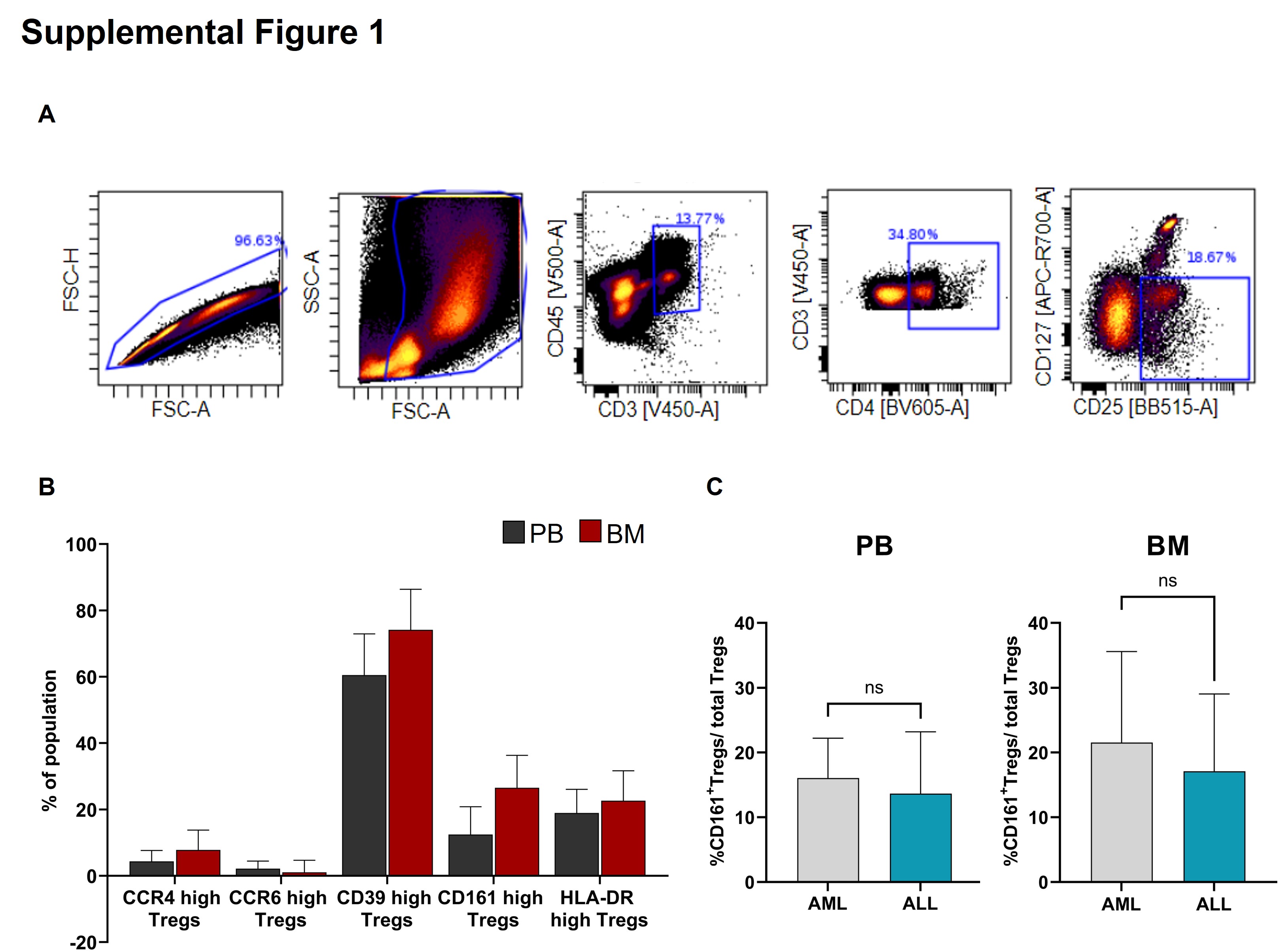

Supplement: Supplementary file 3 — Supplemental Figure 1 [file 41375_2023_1932_MOESM3_ESM.jpg]
