# Supplementary material for: A pro-inflammatory environment in bone marrow of Treg transplanted patients matches with graft-versus-leukemia effect
Source: Leukemia. 2023 Jun 7;37(7):1572–5. doi: 10.1038/s41375-023-01932-x (PMC10317833; doi:10.1038/s41375-023-01932-x)
Supplement: Supplementary file 4 — Supplemental Legend [file 41375_2023_1932_MOESM4_ESM.docx]

**Supplemental Legend**

**Figure 1**

1. Treg gating strategy.
2. Representative histogram of the quantification of CCR4, CCR6, CD39, CD161 and HLADR high Tregs in peripheral blood and bone marrow samples.
3. The percentages of CD161^+^ Tregs in the PB (left panel) and BM (right panel) after 1 month post transplantation were comparable between AML and ALL patients (Student's *t*-test: *p*>0.05). Data are represented as mean ± SD.
