# Supplementary material for: A pro-inflammatory environment in bone marrow of Treg transplanted patients matches with graft-versus-leukemia effect
Source: Leukemia. 2023 Jun 7;37(7):1572–5. doi: 10.1038/s41375-023-01932-x (PMC10317833; doi:10.1038/s41375-023-01932-x)
Supplement: Supplementary file 5 — Supplemental Methods [file 41375_2023_1932_MOESM5_ESM.docx]

**Methods**

***Patients***

Twenty patients (median age, 47; 17 diagnosed with acute myeloid leukemia and 3 with acute lymphoblastic leukemia) transplanted in two centers (*n*=15 at the Ospedale "Santa Maria della Misericordia”, Perugia and *n*=5 at the Ospedale Civile "Santo Spirito", Pescara, Italy) with haplo/Treg HSCT, were enrolled in the present study. Patients transplanted at the Ospedale “Santa Maria della Misericordia” (Perugia) are only a part of the enrolled patients in the clinical trial, registered with the Umbria Region Institutional Review Board Public Registry (code 02/14 and public registry #2384/14) and at [www.clinicaltrials.gov](about:blank) as #NCT03977103 (3) with the inclusion of additional patients recruited at Pescara Hospital. Before enrollment, all patients provided written informed consent in accordance with the Declaration of Helsinki. Patients were recruited in chronological order as they were transplanted and the study was performed only in the last 20 enrolled patients.

Haplo-HSCT with an infusion of 2×10^6^/kg donor Tregs on day −4 followed by 1×10^6^/kg Tcons on day −1 was performed. A megadose of positively purified CD34^+^ hematopoietic progenitor cells was infused on day 0 (1-3). No pharmacological prophylaxis for Graft-versus-host Disease (GvHD) was administered post-transplantation (1-3). Peripheral blood (PB) and bone marrow (BM) samples were collected at 1, 3, 6, and 12 months after transplantation and used for further analysis. This study did not concern the T lymphocytes infiltrating the intestinal mucosa and other GvHD-target organs such as the liver or lung.

**Flow cytometry**

BM- and PB-DCs were analyzed, on a BD FACSLyric™ System (BD Biosciences, La Jolla, CA), using the antibodies listed in Supplemental Table 1. For Treg immunophenotyping and for functional assays the antibodies listed in Supplemental Table 1 were used, dead cells were excluded through 7-Amino-Actinomycin D (7-AAD) staining (#559925; BD Biosciences) or Live/Dead™ Fixable Far Red Dead Cell kit (#L10120; Thermo Fisher Scientific, Waltham, MA, USA), and samples were analyzed on a BD FACSLyric™ System and a BD FACSAria™ III Cell Sorter (BD Biosciences).

For Treg analysis, signal anomalies derived from abrupt changes in the flow rate, instability of signal acquisition and/or margin events, in the lower or upper limit of the dynamic range, were filtered out of individual sample FCS files with the R package flowAI (4). Tregs were manually gated using Cytobank (Supplemental Figure 1) and samples with fewer than 100 gated cells were removed. Then, we applied the Tracking Responders Expanding (T-REX) (5), a novel unsupervised machine learning algorithm for characterizing cells in phenotypic regions of significant change in a pair of samples. Briefly, a UMAP was created using Tregs from all patients and samples (bone marrow and peripheral blood), then each cell was used as the seed for a k-nearest neighbors (KNN, k = 60) search of the local neighborhood within the UMAP axes. When ≥95% of cell’s neighbors belonged to the same sample type (bone marrow or peripheral blood), they were considered a “hotspot” of significant change. Cells in change hotspots were clustered using DBSCAN and characterized using Marker Enrichment Modeling (MEM) (6), which indicates features that are enriched relative to the rest of the population, on a scale from 0 (no enrichment) to +10 (most significant enrichment). Then, the percentage of Tregs expressing the markers identified by MEM was assessed using manual gating. All analyses were performed on a BD FACSAria™ III Cell Sorter System (BD Biosciences).

***Real-time RT-PCR***

DCs were sorted, from PB and BM samples collected at 1, 3 and 12 months after transplant, by using anti-CD123 (for plasmacytoid DCs, pDCs) and anti-CD11c Abs (for myeloid DCs, mDCs) (Supplemental Table 1), and analyzed, by real-time RT-PCR, for the expression of the *Indoleamine 2,3-Dioxygenase 1* (*IDO-1*), *Interleukin (IL)-6*, *IL-10*, *Programmed Death Ligand 1* (*PD-L1*) and *Transforming Growth Factor Beta 1* (*TGFB1*). RNA was extracted from sorted DCs using the RNeasy Plus Micro Kit (#74034; Qiagen, Hilden, D) and cDNA was obtained using the Prime Script RT Master Mix (#RR036B; Takara Bio, Kusatsu, J). Real-time qPCR was performed with PCR Master Mix Power SYBR Green (#4368702; Thermo Fisher Scientific) on a 7900HT fast Real-Time PCR System (Thermo Fisher Scientific, RRID:SCR_018060). Primers for *IDO-1*, *IL-6*, *IL-10*, *Glyceraldehyde-3-Phosphate Dehydrogenase* (*GAPDH*), *PD-L1* and *TGFB1* mRNA (listed in Supplemental Table 2) were designed and synthesized by Sigma-Aldrich Corporation (St. Louis, MO, USA). Relative fold change in gene expression was normalized to GAPDH and calculated using the 2 ^-Δ/ΔCt^ method.

***Dendritic/T cells co-culture***

To study the effects of mDCs on T cell proliferation, pre-activated (with GM-CSF, at 50 ng/ml, IL-4, at 800 U/ml and TNF-alpha, at 50 ng/ml, for 18 hrs) (#300-03 and #200-04; PeproTech, Rocky Hill, NJ, USA; #RTNFAI; Thermo Fisher Scientific, respectively) BM- and PB-CD11c^+^DCs, collected from patients at 1, 3, 6 and 12 months after transplant, were co-cultured for 96 hours with autologous carboxyfluorescein diacetate succinimidyl ester (CFSE, Thermo Fisher Scientific) labeled BM- and PB-CD3^+^ T cells, at a DC:CD3^+^ ratio of 1:10. Subsequently, CD3^+^ T cells were collected and analyzed using a BD FACSLyric™ System.

***In vitro T cell differentiation***

For CD161^+^ Treg cell generation, Tregs purified from PB of healthy donors (*n* = 6 cases) using Tregs isolation kit (CliniMACS® CD8 Reagent, #200-070-215; CliniMACS® CD19 Reagent, #200-070-204; CliniMACS® CD25 Reagent, #200-070-211; Miltenyi Biotec, Bergisch Gladbach, Germany), were seeded in TexMACS™ GMP Medium (#170-076-307; Miltenyi Biotec) supplemented with 5% AB serum (from a local blood bank) and 1000 U/ml IL-2 (#CLB-P-476-800-13980 IT; Novartis, Basel, CH), activated with MACS® GMP T Cell TransAct™ (#170-076-156, Miltenyi Biotec) and cultured up to 10 days in the presence of 5 ng/ml recombinant human TGF-β (#100-21; PeproTech, London, UK) or 25 ng/ml recombinant human IL-6 (#200-06; PeproTech, London, UK) or both, added every 48 hrs. Subsequently, cells were collected at day 10 of culture, and analyzed with a BD FACSLyric™ System using the antibodies listed in Supplemental Table 1. Furthermore, at day 10, Tregs were sorted according to CD161 expression using BD FACSAria™ III Cell Sorting instrument and used for experiments. As control, an aliquot of Tregs cells freshly isolated from the PB of the same healthy donors was seeded in TexMACS™ GMP Medium, supplemented with 5% AB serum, 1000 U/ml IL-2, and 100 nM Miltenyi MACS® GMP Rapamicyn. Tregs were stimulated with MACS® GMP T Cell TransAct™, as already described (7,8). After 10 days of cultures, these cells, referred as expanded Tregs were collected, analyzed with a BD FACSLyric™ System using the antibodies listed in Supplemental Table 1, and used for the experiments.

***Cytotoxicity Assay***

A flow cytometry-based assay was used to assess the killing of K562 (RRID: CVCL_0004, DSMZ, Leibniz Institute, Braunschweig, Germany) by Tcons cells, in the presence or not of autologous CD161^+^ Treg cells and autologous expanded Tregs. Target cells were labeled with 2,5 μM CFSE and seeded in 96-well round-bottom plates. T cons cells and Tregs were added at different Tcons:target ratios and different Tcons-Tregs ratios. After 24 hrs, the viable and CFSE positive target cells were analyzed using a BD FACSLyric™ System. The difference between the viability of the control (K562 alone) and the other samples with Tcons and Tregs was represented as: Killing (%) = [1 – viable target cells (sample) / viable target cells (control)] × 100%.

***Suppression Assay***

The proliferation of Tcons was stimulated with Miltenyi MACS® GMP T Cell TransAct™, and the suppressive activity of CD161^+^ Treg cells and expanded Tregs was assessed by coculturing Tcons, labeled with 2,5 µM CFSE with  autologous Tregs at different ratios. After 96 hrs, CFSE positive Tcons were analyzed with BD FACSLyric™ System, and the suppressive activity was calculated as: [(% proliferating Tcons alone – % proliferating T cons in co-culture with Tregs) / % proliferating Tcons alone] × 100%.

**References**

1. Di Ianni M, Falzetti F, Carotti A, Terenzi A, Castellino F, Bonifacio E, et al. Tregs prevent GVHD and promote immune reconstitution in HLA-haploidentical transplantation. Blood. 2011;117(14):3921-8.

2. Martelli MF, Di Ianni M, Ruggeri L, Falzetti F, Carotti A, Terenzi A, et al. HLA-haploidentical transplantation with regulatory and conventional T-cell adoptive immunotherapy prevents acute leukemia relapse. Blood. 2014;124(4):638-44.

3. Pierini A, Ruggeri L, Carotti A, Falzetti F, Saldi S, Terenzi A, et al. Haploidentical age-adapted myeloablative transplant and regulatory and effector T cells for acute myeloid leukemia. Blood Adv. 2021;5(5):1199-208.

4. Monaco G, Chen H, Poidinger M, Chen J, de Magalhaes JP, Larbi A. flowAI: automatic and interactive anomaly discerning tools for flow cytometry data. Bioinformatics. 2016;32(16):2473-80.

5. Barone SM, Paul AG, Muehling LM, Lannigan JA, Kwok WW, Turner RB, et al. Unsupervised machine learning reveals key immune cell subsets in COVID-19, rhinovirus infection, and cancer therapy. Elife. 2021;10.

6. Diggins KE, Gandelman JS, Roe CE, Irish JM. Generating Quantitative Cell Identity Labels with Marker Enrichment Modeling (MEM). Curr Protoc Cytom. 2018;83:10 21 1-10 21 8.

7. Ulbar F, Villanova I, Giancola R, Baldoni S, Guardalupi F, Fabi B, et al. Clinical-Grade Expanded Regulatory T Cells Are Enriched with Highly Suppressive Cells Producing IL-10, Granzyme B, and IL-35. Biol Blood Marrow Transplant. 2020;26(12):2204-10.

8. Del Papa B, Ruggeri L, Urbani E, Baldoni S, Cecchini D, Zei T, et al. Clinical-Grade-Expanded Regulatory T Cells Prevent Graft-versus-Host Disease While Allowing a Powerful T Cell-Dependent Graft-versus-Leukemia Effect in Murine Models. Biol Blood Marrow Transplant. 2017;23(11):1847-51.
